# Scaling approaches and macroecology provide a foundation for assessing ecological resilience in the Anthropocene

**DOI:** 10.1098/rstb.2023.0010

**Published:** 2024-05-27

**Authors:** Brian J. Enquist, Doug Erwin, Van Savage, Pablo A. Marquet

**Affiliations:** ^1^ The Santa Fe Institute, 1399 Hyde Park Road, Santa Fe, NM 87501, USA; ^2^ Department of Ecology and Evolutionary Biology, University of Arizona, Arizona, AZ 85721, USA; ^3^ Department of Paleobiology, MRC-121, National Museum of Natural History, Washington, DC 20013-7012, USA; ^4^ Department of Ecology and Evolutionary Biology and Department of Computational Medicine, University of California, Los Angeles, Los Angeles, CA 90095, USA; ^5^ Instituto de Sistemas Complejos de Valparaíso (ISCV), CP 2340000 Valparaíso, Chile; ^6^ Departamento de Ecología, Facultad de Ciemcias Biológicas, Pontificia Universidad Católica de Chile, CP 8331150, Santiago, Chile; ^7^ Centro de Modelamiento Matemático (CMM), Universidad de Chile, International Research Laboratory, 2807, CNRS, CP 8370456 Santiago, Chile

**Keywords:** macroecology, resilience, theoretical ecology, disturbance

## Abstract

In the Anthropocene, intensifying ecological disturbances pose significant challenges to our predictive capabilities for ecosystem responses. Macroecology—which focuses on emergent statistical patterns in ecological systems—unveils consistent regularities in the organization of biodiversity and ecosystems. These regularities appear in terms of abundance, body size, geographical range, species interaction networks, or the flux of matter and energy. This paper argues for moving beyond qualitative resilience metaphors, such as the ‘ball and cup’, towards a more quantitative macroecological framework. We suggest a conceptual and theoretical basis for ecological resilience that integrates macroecology with a stochastic diffusion approximation constrained by principles of biological symmetry. This approach provides an alternative novel framework for studying ecological resilience in the Anthropocene. We demonstrate how our framework can effectively quantify the impacts of major disturbances and their extensive ecological ramifications. We further show how biological scaling insights can help quantify the consequences of major disturbances, emphasizing their cascading ecological impacts. The nature of these impacts prompts a re-evaluation of our understanding of resilience. Emphasis on regularities of ecological assemblages can help illuminate resilience dynamics and offer a novel basis to predict and manage the impacts of disturbance in the Anthropocene more efficiently.

This article is part of the theme issue ‘Ecological novelty and planetary stewardship: biodiversity dynamics in a transforming biosphere’.

Resilient responses are emergent properties resulting from component processes of persistence, recovery, and reorganization, with different mechanisms at work in each mode.

 Falk *et al.* [1, p. 1]

Despite their complexity, ecological systems are not haphazard collections of organisms interacting randomly. Instead, they exhibit a great deal of order: in the kinds of organisms that make up the system, like their interactions with each other and their nonliving environment, and especially in the emergent structure and dynamics of the system. This order is perhaps best revealed in certain statistical patterns.

 Brown, J.H. [2, p. 4]

## 1. Introduction

### (a) The parable of the North American chestnut

In the early 1900s, North America witnessed the loss of a dominant tree species of the eastern deciduous forest, the American chestnut (*Castanea dentata*, Fagaceae; [3,4]). For years, the American chestnut tree largely defined American deciduous forests (box 1)—it was a large tree that ranged from Maine to Georgia and west to the prairies [5]. It survived all evolutionary adversaries for 40 Myr, but within 40 years, it effectively disappeared largely owing to a fungal pathogen [19]. The severity of the decline of the American chestnut may be because it had no history of evolutionary interaction with this new exotic fungal pathogen [20]. First discovered in 1904 in New York City, the chestnut blight was a global pandemic hitting forests worldwide. It is estimated that in some places, such as the Appalachians, one in every four trees was an American chestnut [21]. Within a generation, the blight killed approximately four billion chestnut trees, and the forests of North America had effectively no chestnut trees. The blight caused significant tree loss and led to the effective extirpation of a dominant species [19,22,23]. The loss of the chestnut has been called the single most significant ecological catastrophe in forest history [7,24].

Box 1. **What was the ecological impact of the effective sudden loss of a major ecological dominant species (the North American Chestnut)?**

American chestnut was once a dominant or codominant canopy tree over much of eastern North America [5,6]. The chestnut blight, caused by the fungus *Cryphonectria parasitica* [7] brought a dramatic ecological, economic and societal change to the eastern USA that still resonates today [4,8–10]. Originating in Southeast Asia, this pathogen rapidly spread across North America via international travel networks, hitting economically disadvantaged areas particularly hard. The blight's rapid spread devastated the American chestnut tree population, once a dominant species, significantly altering local economies and lifestyles [4].

It was a prolific nut producer and provided an important food source for wildlife. The chestnut wood, valued for its lightweight, soft and decay-resistant properties, was central in constructing American homes and other infrastructure. An estimated 600 million board feet of chestnut were cut in the USA in 1907 alone, translating to a modern retail value of over three billion dollars. Moreover, the chestnut nut harvest offered significant ecological and economic value, contributing to self-sufficient agriculture and providing an essential food source for domestic livestock. The blight's sweeping effects resulted in the loss of an estimated four billion chestnut trees in the first half of the twentieth century. This drastic decline drove rapid ecological and societal shifts. The loss of the chestnut was associated with the transition from subsistence agriculture (partly owing to the loss of the subsistence harvest of chestnut) to industrialization, becoming particularly prominent in Appalachia [7].

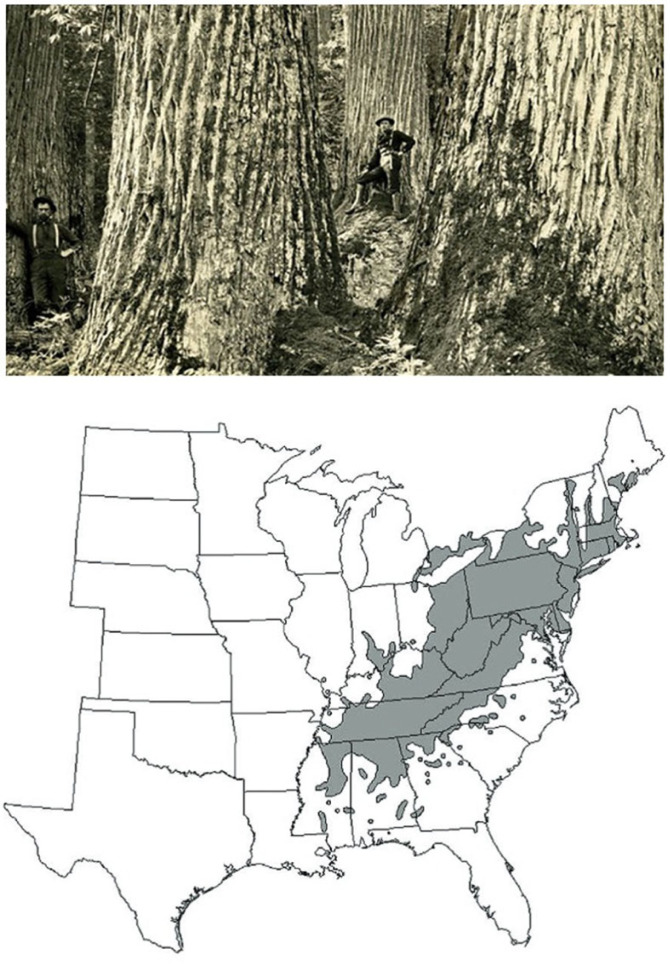

*The cascading ecological impacts of the loss of the American chestnut*: the ecological impacts of the sudden loss of the chestnut are unclear. Still, the indication was that it was extensive [7]. Depending on the local environment and soil type, different tree species replaced the dominance of chestnut. Woods & Shanks [11] reported that in the Great Smoky Mountains National Park, 41% of all replacements of chestnut were oak species. They found that pure hemlock stands (*Tsuga canadensis* (L.) Carr.) replaced chestnut in some areas. Nelson [12] reported that tuliptree (*Liriodendron tulipifera* L.) was the most frequent replacement species in western North Carolina, significantly changing such sites' ecology. Chestnut blight probably altered nutrient cycling. Chestnut wood is slower to decay than other hardwoods, such as oak species associated with it [13]. However, chestnut leaves decay more rapidly and have higher nutritional value than oak. As a result, the shift in species composition probably shifted, and the nutrient status of soils may have declined [14]. This shift in species dominance and cascading impacts shifted the species composition, diversity, and ecology of eastern deciduous forests. Loo states, ‘The forest that replaced chestnut has a greater diversity of tree species, so local biodiversity may have increased as a result of the disease [15], but the functional loss of a species with such ecological dominance undoubtedly influenced species diversity at a variety of trophic levels, including mast-dependent birds and mammals and their associated predators, parasites and pathogens, as well as soil micro flora and fauna as a result of the loss of chestnut from eastern hardwood forests’ [7, p. 87].

Despite the massive impact of chestnut blight, did eastern deciduous forests and ecosystems exhibit resilience and recover? Unfortunately, the true extent of the impacts may never be fully understood owing to the lack of quantitative data [7]. However, no species appears to have been set to replace the exact ecological function once performed by the chestnut. The question remains: have the affected forest communities and ecosystems achieved a comparable or novel ecological baseline? Subsequent studies indicate that forest species and biodiversity have shifted since the loss. Still, according to de Liocourt's Law, species abundance and size distribution analyses suggest that these communities have returned to similar macroecological baselines [16].

This picture, taken in the mid-to-late nineteenth century, shows how large and profuse the American chestnut tree was in eastern United States forests. The blight killed in a relatively short time span approximately four billion chestnut trees. (Courtesy photo American Chestnut Foundation; https://acf.org/.) See https://www.usda.gov/media/blog/2019/04/29/what-it-takes-bring-back-near-mythical-american-chestnut-trees. The native range of the American Chestnut (*Castanea dentata*, Fagaceae) in eastern North America. Range map from Jacobs [17] as adapted from [18].

What was the ecological impact of the effective loss of a major ecological dominant species? As discussed in box 1, the loss of chestnut caused a series of cascading impacts [4,13]. In visiting eastern deciduous forests today, one would be hard-pressed to conclude that the ecology and biodiversity of North America witnessed a massive ecological perturbation and loss. While different in dominance and species composition, today's forests seem ecologically vibrant, biodiverse and structurally similar to other forests [16,23]. Nonetheless, it is unclear what the ecological impact of this loss has been on forest ecology and ecosystem functioning [4,13]. Indeed, the case of the American chestnut underlines the need to define ecological baselines and develop a more predictive ecological science that can (i) understand how escalating ecological disturbances impact and shape biodiversity interactions and ecosystem functioning, and (ii) more accurately predict what determines ecological resilience and ecosystem recovery.

### (b) Ecological resilience in the Anthropocene

Rooted in C.S. Holling's seminal work [25], the concept of resilience, along with its related constructs of stability and robustness, has broadened its scope beyond mere ecological dynamics to embrace the intricate interplay within socio-ecological systems (e.g. [26–28]). Stability is widely recognized as a system's inherent ability to revert to its initial state post-alteration, robustness is seen as the capacity of a system to resist changes in its state or functionality upon encountering a disturbance, and resilience is characterized by the system's ability to attain a state of comparable functionality after undergoing change [26,29,30].

Today, ecosystems face unprecedented challenges. Under the escalating forces of climate change and anthropogenic pressures, it is unclear how resilient ecosystems and biodiversity are to increasing change and disturbance. A notable consequence of the Anthropocene is the displacement of key climatic variables beyond their historical ranges of variability, pushing species and ecosystems into novel conditions, as well as novel interactions with each other, and posing new threats and challenges [31,32]. Such shifts bring to the forefront of a critical aspect of resilience research: what ecosystem components—be they species abundance, functional metrics, or biodiversity—are crucial for the focus of restoration and recovery efforts in the aftermath of a disturbance [1]? Addressing this question demands an integrative approach that melds comprehensive, comparative and macroecological perspectives to fully grasp and apply the concept of ecological resilience [1,33]. Further, to understand the impacts on disturbed and undisturbed communities, it is essential to define clear baselines for comparison [34]. This approach helps prevent the ‘shifting baseline syndrome’, a phenomenon where baselines are inaccurately established based on short-term observations, leading to a misrepresented norm that reflects a progressively degraded state over time [35].

A critical aspect of contemporary ecological research is deciphering how complex ecological systems respond to the escalating frequency and magnitude of disturbances [36]. Do these systems maintain their inherent structure and functionality, or are they driven to transform [37]? Are there thresholds beyond which resilience is compromised [38]? Such changes are expected to trigger a cascade of physiological and behavioural adaptations defining ecological interactions among species [39,40]. For example, on ecological timescales, a local disturbance—a pathogen outbreak, a fire, a severe drought, etc.—could trigger a series of population shifts. These shifts are characterized by altered mortality and recruitment patterns within and across species as some individuals adapt, grow and recolonize the affected areas. Over time, these differential demographic responses, encompassing birth, death and migration processes within and between species, will probably manifest as significant ecological changes. These changes are reflected in the post-disturbance growth trajectory dynamics of the assemblage of species.

A more fundamental and pressing question is whether disturbances lead to ecological reorganizations that align with general patterns of recovery and function or diverge into idiosyncratic and diverse pathways that substantially shift ecosystem functioning (e.g. [41,42]). If the former holds, it suggests the potential for a predictive theory of resilience. The latter would indicate that our future is much more idiosyncratic and uncertain in the face of increasing global change though we still might be able to identify tipping points, points of no return, and management strategies as we approach such an idiosyncratic set of dynamics.

## 2. Moving beyond the ‘ball and cup’ heuristic of ecological resilience

**Figure 1.**
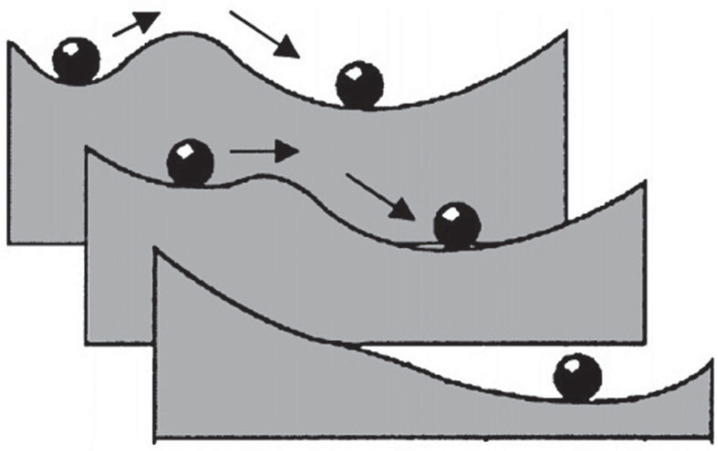
Diagram from Gunderson (2000) shows a ball and cup heuristic of system stability. Valleys represent stability domains, balls represent the system and arrows represent disturbances. Adaptive capacity refers to the ability of the system to remain in a stable domain as the shape of the domain changes associated with a change in a controlling variable or parameter (as shown by the three slices or landscapes).

Resilience is often conceptualized and associated with ‘ball and cup’ diagrams or adaptive landscapes [26,37]. These intuitive graphical analogies seemingly convey how various forces can drive ecological systems to certain states (see [37]). The ball represents the system state, and the cup represents the stability domain or basin of attraction associated with a given state (figure 1). An equilibrium exists when the ball sits at the bottom of the cup. Perturbations shift the system to a transient position and potentially to a new state. Such diagrams have helped conceptualize how resilience (and stability) can be measured by the magnitude of disturbance absorbed before the system shifts to a new state by changing the variables and processes that control behaviour. In this context, the equilibrium will be stable if the perturbation is small enough not to displace the system to the alternative equilibrium, and resilience will be a measure of that phenomenon. However, what do we mean by equilibrium Or even alternative new state?

Since the seminal work of Lewontin [43], the possibility for alternative stable states and transitions between them has been a recurrent topic (e.g. [44]). Alternative stable states are central to our understanding of resilience—and particularly what Holling called ecological resilience [45]—emphasizing the capacity of a system to absorb disturbance and reorganize to retain identity and structure (see [46,47]). This approach to resilience envisions ecological systems as moving in stable landscapes in response to the interaction between internal and external or environmental changes in processes and structure. This landscape is populated by alternative local equilibrial states that may satisfy the definition of ecological resilience given above. The issue is how can we know that the system has been reorganized to maintain identity and structure? In this contribution, we argue that macroecology offers the potential for a unique framework for advancing the study of ecological resilience, providing empirically tractable ways to begin assessing the space of viable and persistent reorganizations of systems in the face of disturbance.

We draw from prior studies [48–53] and explore how macroecology, with its emphasis on broad patterns [54,55], coupled with a theoretical and scaling approach [1,48] can provide a novel and robust framework for reformulating studies of ecological resilience. The Anthropocene presents numerous challenges that demand not only a general first-principles theory to predict changes (e.g. [56]) but also a set of methodological tools to assess ecological dynamics in response to disturbance [50]. The premise of macroecology is that at some level of analysis, ecological systems exhibit strong, emergent regularities and that aspects of these distributions (e.g. the distribution of abundance, body size, geographical range size, traits etc., observed in ecological assemblages of species and individuals) ultimately govern the seemingly complex and idiosyncratic behaviour of ecological dynamics [2,48,57,58]. In this sense, we argue that the focus of resilience studies should move beyond traditional measures of stability, persistence and resilience that focus on the particular abundance or attribute associated with each species and instead focus on more macroecological measures that focus on how emergent properties of the assemblage (such as abundance or size distributions) and how these properties may shift with perturbation.

### (a) Hypothesis: macroecological patterns are a strong baseline for resiliency studies

In 1898, François de Liocourt published a manuscript in *De l'amenagement des sapinières Bulletin trimestriel, Société forestière de Franche-Comté et Belfort* entitled ‘The management of silver fir forests’ [59]. He observed that in untouched ecological communities, the size distribution of organisms typically forms a ‘J-shaped’ pattern, signifying an uneven age and size structure. De Liocourt posited that disturbed forests tend to return to size distributions akin to those in unmanaged forests, as shown in the bottom curve of figure 1 (from [60]). Thus, the size distribution was hypothesized to reflect a ‘baseline attractor’ from which ecological dynamics could be specifically understood and the impact of disturbances quantified [61].

As discussed below, we assess the generality of *de Liocourt's Law of Resilience* [62] and the more general hypothesis that *the regularities embodied by macroecological distributions often reflect emergent equilibrial ‘*stable state*’ dynamics. As such, they provide a baseline for understanding change and reorganization in ecological systems* [48,51,57]. *A corollary is that deviations from macroecological patterns are a quantitative measure and signature of specific structuring processes that can indicate the transient reorganization of the system and the eventual recovery of the same relationship after disturbance* (e.g. [57] a signature of resilience). A key prediction is that an ecological response to disturbance is predictable around a baseline macroecological distribution.

Not aware of the earlier work of Liocourt, ecologists have also focused on size distributions of animal and plant populations as a general macroecological pattern [63]. The inverse relationship between population density and body size—often referred to as Damuth's rule or the energetic equivalence rule (EER) [64]—within trophic groups emerges from the constraints on individual metabolism and the scaling of the use of space and resources with body size (e.g. [64,65]). The size distribution is an important measure of ecosystem functioning related to total ecosystem stocks and fluxes influencing system biomass and productivity [66]. Scaling relationships of individuals thus give rise to a scaling relationship between population abundance (*N*) and body size (*M*) that can be described as

2.1 $$N=aM^{-b}.$$

An early example of macroecology as a baseline attractor for ecological dynamics comes from Marquet *et al*. [57]. Marquet *et al*. showed that in intertidal communities under strong disturbance owing to human exploitation, the species interaction networks and the abundance of dominant species changed in response to disturbance. Nevertheless, the impacted communities remarkably showed *the same inverse size abundance density scaling* as in sites where humans were excluded (see also [67]). Their results suggested that the intertidal communities demonstrated resilience to human impact in terms of the size structure. They also conclude that ‘…after a perturbation, species within each community (inside and outside either marine preserve) reassemble in a way the net result of which is the same population density scaling. This points to the operation of density compensation phenomena in which some species decrease in density while others increase, coupled with body size shifts’ [57, p. 1126]. This example is consistent with the hypothesis that general emergent macroecological patterns reflect ‘steady state’ or ‘equilibrial’ baselines or ‘attractors’ of ecological dynamics, signalling the reorganization of ecological systems. A signature of resilience would be if, after disturbance, the structure of ecological assemblages returns to general patterns of abundance and size structure even if the system changes the exact composition of in species' identities and abundances. By contrast, permanent deviations from the macroecological baseline distribution pattern could signal a loss of resilience and a shift to a new ecological state.

## 3. Towards a general dynamical scaling theory to assess the resiliency of ecological assemblages to increasing disturbances

We propose a conceptual and theoretical basis of ecological resilience that can be advanced by integrating macroecology with a general theoretical framework based upon a stochastic diffusion approximation. This approach focuses on sufficiently general patterns that do not depend on species identities *per se*. Thus, they can be used to compare communities in different biogeographic regions or ecosystems. The impacts of disturbance on a population or assemblage of species are measured by how they impact age- or size-dependent population and species community dynamics, especially in terms of changes in birth and death rates. Our approach is anchored in three assumptions:

(i) the processes of birth and death (i.e. growth, mortality, immigration, emigration, speciation, extinction etc.) form a foundation for characterizing ecological dynamics and linking many biological processes [68,69]. Specifically, in a demographic context, disturbances can be defined as any external, discrete (a)biotic event that impacts the structural composition of a given population [70] or across species in a given community or assemblage;
(ii) these intra- and interspecific ‘birth and death processes’ are often constrained by a steady demographic state. This biological symmetry in birth and death reflects a balance in the rates and patterns of individual and species turnover, proliferation and demise [71,72]; and
(iii) biological scaling laws profoundly influence birth and death rates (e.g. how individual and species turnover, proliferation, and demise) as these rates all scale with a key trait—organismal body size—and ramify across different levels of ecological organization [73]. Shared, approximately universal, allometric scaling constants and exponents (another form of biological symmetry) within and among species reflect uniformity in how metabolism, form and function scale within and between taxa [74]. These similarities lead to a pronounced similarity in the size-density distributions of communities, that is, independent of species composition, and influence the dynamics of ecosystems [75,76].

We start by noting that ecological systems are inherently open and dynamic. The boundaries set by observers define them, whether a vast tropical forest or a small plot in an intertidal zone. Within these defined boundaries, the focal community of individuals and species interacts with its surroundings. Its dynamics are driven by ‘birth and death processes—such as internal birth and death processes (demography and ecological interactions that influence the per capita growth and survivorship) and external birth and death processes (immigration from the external environment, external sources of mortality). The approach we now present builds on literature that recognizes the importance of diffusion approximation [68,69,77]. This perspective allows us to model the proportional abundance of species within these systems, by drawing parallels between the equilibrium distributions of individuals or species or genes within local populations [69]. Such diffusion approximations also illustrate how deeply interconnected ecological and genetic processes are, predicting equilibrium states that are a product of ‘birth–death’ processes and interactions with the external environment.

The roots of an open system approach stem from work by Andrey Kolmogorov. Kolmogorov was working on the diffusion problem starting from the theory of discrete-time Markov processes [78]. He sought to develop a theory of continuous-time Markov processes and developed both forward and backward versions of discrete-time Markov processes. He applied them to many areas of biology. The forward equation is also known as the Fokker–Planck equation [79], after Adriaan Fokker and Max Planck, who also described it in 1914 and 1917. Applied to ecology, we start with *n*, the initial population size, and *β* the rate of a specific process related to the per capita birth and death rates (e.g. of individuals in a population). The rate of change in population size ${\;p}_{n}^{\prime }(t)=d/dt\;pn\;(t)$ is

3.1 $${\;p}_{n}^{\prime }(t)\;=(n-1)\beta p_{n-1}(t)-n\beta p_{n}\;(t).$$

This equation is used to describe the discrete-time evolution of a population in a birth and death process, where $p_{n}=Pr\;(N(t)=n)$ is the probability that the population has size *n* at a given time, *t* [80]. The first term is the rate at which the population in state *n* increases owing to births from state *n* − 1*.* The second term represents the rate at which the population in state *n* decreases owing to deaths or transitions to other states.

A more general equation, the Fokker–Planck equation [79], describes the time evolution of a probability density function *P*(*x,t*) for a continuous stochastic process in continuous time and space. This equation can be applied to model how the probability density of a population or species' presence changes over space and time. The general form of the equation is

3.2 $$\frac{\partial P(x,t)}{\partial t}=\sum_{i}\frac{\partial }{\partial x_{i}}(A(x|i))P(x|i,t)+\frac{1}{2}\sum_{i,j}\frac{\partial^{2}}{\partial x_{i}\partial x_{j}}(B(x|i,j)P(x|i,t)),$$

where *P*(*x*,*t*) is the probability density function of the process at time *t,x* and represent the state of the process. In ecology, *x* represents a spatial coordinate within a habitat or ecosystem. The first summation term on the right-hand side accounts for the deterministic drift of the process, A(x|i). The second summation term describes the diffusion of the probability density, where B(x|i,j) is the diffusion coefficient of the process, and *i* and *j* are indices denoting the possible states of the process. This term represents the spreading or diffusion of probability density owing to randomness or stochasticity. Note that the term ‘drift’ here is engineering/physics lingo but is the opposite mathematical term and conceptual meaning of ‘genetic drift’, which is pure randomness/stochasticity instead of pure determinism. The term ‘drift’ in the context of engineering and physics refers to a deterministic process that predictably influences the direction or state of a system. ‘Genetic drift’ denotes a stochastic or random process that impacts the frequency of alleles within a population. Unlike the deterministic ‘drift’ in physics and engineering, genetic drift arises from the random sampling of alleles during the formation of offspring. This randomness can lead to significant changes in allele frequencies over generations, especially in small populations, irrespective of the alleles' influence on fitness. When the general form of the equation is applied to evolutionary processes, the process of genetic drift corresponds to the next second-derivative term, and the first-derivative term is selection.

The first term on the right side **of equation (3.2)** describes the tendency of the process to move in a certain direction. The second term represents the diffusion of the process, which describes random fluctuations. This pure birth stochastic process is a jump process because the system changes in discrete states (number of individuals) over continuous time. However, if the state space of the system is continuous, for example, if we work with size changes (as in de Liocourt's Law), then the probability density that the mass or diameter (*x*) of a tree lies between *x* and *x + dx* at time *t* (given that it is *y* at time 0), can be expressed as *ϕ*(*y,x,t*). As shown by Kimura [81,82], the probability distribution of sizes will then change according to the Kolmogorov forward equation:

3.3 $$\frac{\partial \phi (y,x,t)}{\partial t}=\frac{1}{2}\frac{\partial^{2}}{\partial x^{2}}(V_{\Delta x}\phi (y,x,t))+\frac{\partial }{\partial x}(M_{\Delta x}\phi (y,x,t)),$$

where *y* is constant but *x* is a random variable, *M* and *V* are the mean and variance of the amount of the change of the frequency of *x* per generation, and δx, the amount of change in *x* per unit time. If we consider the process retrospectively, that is, if we ask which *y* values are compatible with a given *x* at time *t*, we have to fix *x* at time *t*. Then *y* becomes a random variable, and the equation that describes the evolution of *ϕ* is now called the Kolmogorov backward equation, with the sign of the first-derivative term changing from negative to positive. Still, the sign of the second-derivative term remains unchanged.

The Kolmogorov forward equation is a fundamental tool in studying stochastic processes and is used in various fields, including physics, chemistry, biology, finance and engineering. The diffusion approximation approach of Kolmogorov, has been extended across biology and used in population genetics (e.g. [82,83]). The equation can also be used to model how the frequencies of different genetic variants change over time owing to various factors such as mutation, selection, migration and genetic drift. This can provide insights into the mechanisms of evolution and the dynamics of genetic diversity within populations. It has also been used in population dynamics (e.g. [84] and, more recently, in macroecology (e.g. [85,86]) to study the dynamics of species assemblages). As discussed below, we can also use this approach to understand the dynamics of macroecological distributions, such as size and species abundance distributions or individual size distributions [86,87].

### (a) Birth, death and symmetry in ecological systems

Next, we introduce the importance of symmetry-generating processes as a basis for understanding the processes underlying ecological resilience. These processes place biological constraints on the diffusion assumption. Doing so enhances our predictive ability on ecological dynamics and our understanding of resilience. There are three sources of symmetry in ecology.

First, as stated in Marquet [72], the concept of birth, death and symmetry in ecological systems leads us to revisit the importance of mechanisms like fitness equalization and niche partitioning. These mechanisms—often manifesting as trade-offs between traits like dispersal capacity and competitive ability—regulate population and species-level birth and death rates, introducing a symmetry in species' interactions with their environment [72,88]. This symmetry, pivotal for species coexistence, profoundly influences ecosystem dynamics [72,89].

Stochastic models of species richness, such as MacArthur & Wilson's model [90] and its successor, the Unified Neutral Theory of Biodiversity and Biogeography by Hubbell [88] underscore this symmetry [86]. Based on species birth-death processes, these models have been pivotal in shaping our understanding of species diversity patterns and ecological dynamics. Despite apparent species and individual differences, they propose that species are variations of a fundamental theme [91] in which species have the same probability of dying, reproducing and dispersing, as reflected by their birth and death rates. These theories assume a universal demographic symmetry (often called neutrality, meaning neutral with respect to fitness) at the macroecological and macroevolutionary scales, resonating with the power laws observed in nature that we discuss next [71,92].

Second, the well-documented allometric scaling relationships in biology [74,93] introduce an additional symmetry dimension in ecological systems. Despite many differences between species, species tend to follow similar allometric scaling rules underlying their growth and metabolism [94–97]. Similarities in scaling metabolism and growth impose constraints on birth and death dynamics. They are reflected in constraints on species sizes, diversity and abundances with differing life-history strategies [98,99]. These scaling constrains or trade-offs do not imply strict demographic neutrality (or equivalence or symmetry) in terms of absolute birth and death rates, but they do imply symmetries in terms of relative rates of birth and death across species.

Third, there is a limit on the total biomass and metabolism per unit area per unit time. This limit, named the ‘energetic equivalence rule' or EER, suggests that there is typically a system-specific cap on energy use per unit biomass that is universal across species and individuals, within that system, irrespective of individual size differences [65,71,98]. This rule implies a uniform limit on total resource consumption across all species, indicating a ‘zero-sum dynamic’—an energetic or metabolic symmetry—in how resources and energy are used within ecosystems [92]. Like the neutral theory's assumption of constant total system abundance even as there are potential shifts in species composition and the abundance of each species, the EER maintains that at equilibrium, an ecosystem's total resource consumption matches the supply of limiting resources [65]. Essentially, any increase in an organism's resource use necessitates a corresponding decrease by others, enforcing a metabolic balance or energetic equivalence within the ecosystem [100,101]. These principles, rooted in the fundamental laws of thermodynamics and resource distribution and additional ecological assumptions [65,74,102,103], provide a cohesive framework for understanding the underlying energetic symmetry in ecological systems [65,66,76,99,104,105].

### (b) Abundance distributions

Applied to a focal ecological community, species richness and abundance dynamics can be modelled as birth and death processes and immigration from the outside. Marquet *et al*. [86] analyse the distribution of species abundances as a continuous process. They start with *N_J_*(*t*), the number of living individuals of a given species within a focal community of size *J*, at time *t* ≥ 0. Marquet *et al*. model the dynamics as a birth and death process characterized by the transition matrix P(*t*) = (*P_n,m_*(*t*);*n*,*m* = 0, … ,J), where *n* and *m* denote the number of individuals. For a small time increment *h* and *n* ≥ 0, the entries of this matrix satisfy the following as *h* → 0:

3.4 $$\begin{array}{c} \;\\ \begin{array}{cc} P_{n,n+1}(h)=B_{J}(n)h+o(h),\; & \mathrm{for}\;n\geq 0,\;\\ P_{n,n-1}(h)=D_{J}(n)h+o(h),\; & \mathrm{for}\;n\geq 1,\;\\ P_{n,n}(h)=1-(B_{J}(n)+D_{J}(n))h, & \mathrm{for}\;n\geq 0\\ P_{n,m}(0)=\delta_{n,m,}\; \end{array} \end{array}\}.$$

Here, *B_J_* (*n*) and *D_J_* (*n*) represent the ‘birth and death rates’ of species within the focal community (for example, species immigrating into a community and species emigrating or becoming locally extinct within a community), respectively.

Considering the asymptotic behaviour with the diffusion approximation as the community size *J* becomes very large, Marquet *et al*. [72,86] show that the rescaled birth and death rates are

3.5 $$\begin{array}{rl} \underset{J\to \infty }{\lim}\;b_{J}(x_{J}) & =b(x),\;(x\in [0,1])\\ \mathrm{and}\;\;\;\;\;\;\;\;\;\underset{J\to \infty }{\lim}\;d_{J}(x_{J}) & =d(x),\;(x\in [0,1]) \end{array}\}.$$

The rate *c_J_*, representing interactions or migration, is expected to vary with *J*. Marquet *et al*. show how the Fokker-Planck equation can be used to predict the steady-state probability density *ρ_t_* (*x*) of the species abundance distribution.

Applied to species abundance distributions, when *x* is the proportion of individuals in a given species, it has been proved that using a diffusion approximation, if birth and death rates are symmetric (following the functional form used in neutral theory [106,107]) the stationary distribution for the species proportional abundance distribution, $\rho_{\infty }\;(x)$, (the same as for genes [86]), is a beta distribution. The parameters *α* and *β* quantify the relative importance of immigration and speciation, respectively, in relation to stochastic fluctuations. Symmetry (neutrality) satisfies a Fokker–Planck equation whose stationary solution, $\rho_{\infty }$, or invariant distribution, is a beta distribution [86] where

3.6 $$\rho_{\infty }(x)=\frac{\Gamma (\alpha +\beta )}{\Gamma (\alpha )\;\Gamma (\beta )}x^{\left(\alpha -1\right)}{\left(1-x\right)}^{\beta -1}.$$

Here, *α* and *β* are the shape parameters of the beta distribution and are given by variation in species birth (immigration) and death (local extinction) (see Marquet *et al*. [86]). Together, they determine the distribution shape, influencing how the probability is distributed across the range of *x*. It can predict how changes in species' birth and death rates will affect species' overall abundance distribution and provides a basis for how perturbations will impact *ρ_t_*(*x*) [86]. Different values of *α* and *β* can make the distribution skewed in various ways, symmetric, or take on other shapes.

The prediction provides good fits for species abundance distributions when abundance is expressed as a proportion (i.e. the proportional abundance distribution), including those distributions that exhibit power law scaling whenever *α* or *β* equals 1 [86]. *A priori* knowledge of birth and death rates of a given species assemblage and how specific disturbances would then potentially shift these rates would enable specific predictions for how the abundance distribution will change but also the time course of a return to a similar or different steady-state distribution. Also, this version is expressed in terms of gamma functions, which are generalizations of factorials to non-integer values.

### (c) Size distributions

Diffusion approximations can also be applied to the dynamics of another macroecological distribution—size distributions [87,108,109]. The size distribution results from two dynamic processes: individual (birth) recruitment and growth and individual (death) mortality. In a diffusion model applied to a forest size distribution, we treat the size of a tree as a ‘particle’ undergoing a ‘random walk’ owing to growth, *G*(*D*), and variance in growth rates, *V*(*D*). Over time, trees ‘diffuse’ from smaller to larger size classes, akin to particles spreading out in space. A forest's steady-state distribution (or SSD), describes its structure at a point in time but results from the dynamic processes of stem birth (recruitment, growth) and stem death (mortality).

The recruitment dynamics, *R*. This process introduces new stems into the smallest size class within the forest. The size-specific growth process, *G*(*D*), governs the rate at which individual stems advance through different size classes. It is a function of the stem diameter, *D*, indicating that growth rates vary with size. Finally, the size-specific mortality, *M*(*D*), represents the rate at which stems of a particular size dies each year. Like growth, mortality is also a function of stem diameter.

When these processes—recruitment, growth and mortality—operate consistently over a sufficiently long period, and if the total population size remains stable, the forest will probably reach an SSD [87,108,109]. This SSD can be described using a partial differential equation, specifically a forward Kolmogorov equation:

3.7 $$\frac{1}{2}\frac{\partial^{2}}{\partial D^{2}}[V(D)N(D)]+\frac{\partial }{\partial D}[G(D)N(D)-M(D)N(D)]=0.$$

The forward Kolmogorov equation, in this context, captures how the probability density function of tree sizes changes over time. Here, *N*(*D*) denotes the number of stems at a specific diameter *D*, *V*(*D*) represents the variance in growth rates at diameter *D*, and *G*(*D*) and *M*(*D*) are as defined earlier. This equation effectively captures the equilibrium state of forest structure as influenced by its intrinsic biological processes.

Modelling the SSD of a forest as a diffusion problem with the forward Kolmogorov equation provides a robust framework for understanding and predicting how forests change, underpinning both theoretical ecology and practical forest management. For forest management, understanding the SSD helps predict the future structure of the forest and how to plan conservation and harvesting strategies. It can offer insights into how disturbances (like fires or diseases) might shift the steady state, affecting forest resilience and biodiversity.

A shortened version of the above equation relates the relative change in population size, ln *N*(*D*), with the relative change in tree diameter, ln *D*. A change in size in a community relates the size-dependent mortality to the growth of trees, essentially showing how growth and mortality rates interact as a function of size:

3.8 $$\frac{\partial \;\mathrm{In}\;N(D)}{\partial \;\mathrm{In}\;D}=\frac{DM(D)}{G(D)}+\frac{\partial \;\mathrm{In}\;G(D)}{\partial D}.$$

Here, *N*(*D*), is the function for the number of stems of a given diameter, *D*, and *M*(*D*) is a mortality rate function, showing how mortality rates change with tree diameter, *D*. Equation (3.8) shows the balance between growth and mortality in determining the distribution of sizes within an ecological community. The left side (change in the number of individuals with size) is balanced by two forces on the right side: the direct effect of size on mortality and growth rates and the rate at which the growth rate changes with size.

By itself, the Kolmogorov equation (equation (3.8)) is arguably phenomenological. At steady state, the size distribution *must necessarily result from* size-dependent growth and mortality [110]. A diffusion approach does not predict the birth and death processes—it does not predict either size-dependent growth or mortality. Instead, it uses these as input parameters to generate the emergent distribution (here, specifically, the size distribution). Nonetheless, the diffusion approximation shows us that stochastic birth and death processes must reach an emergent steady state at the larger time and spatial scales. As such, it provides a set of predictions for the emergent pattern and timescale of how an assemblage reaches its steady-state structure from a given initial condition [111]. The Kolmogorov equation does *not* provide a mechanistic basis for the origin of size spectra or size distributions. The mechanistic origin is based on the symmetry of processes invoked by metabolic scaling theory. Ultimately, metabolism fuels growth and powerfully constrains plant form, determining the size distribution and scaling of competitive mortality [99,105].

We can impose symmetry into the Chapman–Kolmogorov equation (equations (3.2) and (3.8)) from the zero-sum resource use assumption [65,98,99,105] and by including the observation that the rate of change of a biological ‘trait’ *x* is probably dependent on age, body size and other aspects of biological scale (area, time and temperature) according to allometric scaling functions that are similar across species [73]. Thus, biology can be incorporated via scaling functions defining recruitment, growth and mortality [99,105] and imposing zero-sum metabolic constraints, see box 2. Specifically, population demography (probability of birth and death) is a function of organismal size [120]. The moments *V* and *M* that describe the dynamics *δx* can be parameterized regarding scaling functions from metabolic scaling theory [74]. For example, the left side of equation (3.1) represents the log-log SSD's slope, which, according to metabolic scaling theory [76,99,105], should be −2 (see box 2) when size is measured in terms of stem diameter, *D*. Thus, theory predicts a steady state (symmetrical) size distribution (de Liocourt's law). The left side represents the log-log SSD's slope, which, at resource steady state, is −2. This implies a specific symmetry or balance on the interplay between the scaling of growth and the scaling of mortality expressed as

3.9 $$\frac{M(D)}{G(D)}=2-\frac{D}{\partial \;\mathrm{In}\;G(D)/\partial D}.$$

Box 2. **de Liocourt's Law of Ecological Resilience as a general approach to using scaling and macroecological patterns.**

de Liocourt's law states that in a resource use and demographic steady state, the number of individuals as a function of their size will reach an approximate steady-state distribution. This distribution is named after the French forester Francois Henri de Lallemant de Liocourt's 1898 paper. It is an example of using a macroecological pattern to quantify and assess how a perturbation influences a population, an ecological community, or an ecosystem and how resilient that system is to a given perturbation. Although originally applied to uneven-aged forests, de Liocourt's law can be generalized to size distributions (of plants and animals). Further, similar insights could also be inferred from applying similar dynamical ‘steady state’ thinking to other patterns or distributions in ecology, such as species rank abundance or species abundance distributions, species-area curves, and the distribution of traits or behaviours. Each of these potential distributions has a long literature, and there is an understanding of the generality associated with these patterns and the degree of variation (e.g. [55,112–114]). Focusing on these patterns or a suite of these patterns enables one to characterize the baseline distribution for a given location and to understand the natural variation owing to climate and other potential environmental drivers.

In this example [59,60], the distribution of organismal body size follows an inverse relationship where there are more small individuals than large ones. The theory provides predicted mathematical functions for size frequency distributions [76,87,99,105,115]. According to this literature, within a given ecological community, all individuals will be governed by three general principles:

(i) all individuals follow similar allometric scaling rules for metabolism and growth [74,102];
(ii) within ecological communities, there is a strong selection for individuals to fill all available space to use all available resources [65,99]; and
(iii) those individuals that have traits that allow them to perform (grow, survive according to (i)) and reproduce best will tend to increase in abundance and tend to become more dominant [116].

As a result of principles (i)*–*(iii), the size distribution will begin to approximate a demographic steady state with a specific functional form that reflects two fundamental biological phenomena: metabolism and allometry. Together, they determine how resources are taken up from the environment, translocated and transformed within organisms, and allocated to survival, growth and reproduction [117]. In support of these predictions, analyses of size distributions in differing environments show similar functional forms *f*(*m*) with similar exponents, especially in the absence of disturbance [16,76].

The distribution can be characterized by either a Pareto distribution or a truncated Pareto distribution (due to an upper limit on organismal size) [118]. For the Pareto distribution, we have

5.1 $$f\;(m)=-(\lambda +1)m_{0}^{-(\lambda +1)}m^{\lambda }.$$

Here, *f*(*m*) is the frequency of individual sizes *m*, which can range from *m*_0_, the minimum size of individuals varying within a given site or across sites to the largest. The scaling exponent, *λ*, describes the change of abundance with mass. However, the Pareto distribution does not consider a possible limit on maximum size. As predicted by metabolic scaling theory, resource limitation places an upper limit on maximum size, *m*_max_ [105]. As a result, the distribution of sizes should ‘bend down at the largest sizes, where

5.2 $$(m)=(\lambda +1)\;(m_{\max}^{\left(\lambda +1\right)}-m_{0}^{\left(\lambda +1\right)})m^{\lambda }.$$

The exponent's value reflects much biology and potential differences between populations and species. Specifically, *λ* is determined by demography (how individuals grow and die as a function of their size, *m*) and how individuals fill space and compete with each other (see [76,87,99,105,115,119]). Thus, we can move beyond the ‘ball and cup’ analogy for the study of resilience (figure 1) and instead quantify the impacts of perturbations and resilience in the temporal dynamics of the variables that define macroecological functions. In the case below, for any given assemblage of organisms, we can characterize the distribution of individuals via *λ*, *m*_max_ and *m*_0_. In doing so, it is important to emphasize that much biology underlies what sets the values of *λ*, *m*_max_ and *m*_0_, in any ecological assemblage.

In the example below, we can use the distribution of sizes pre-perturbation (*a*) as a baseline to assess how a perturbation influences the assemblage and how the assemblage of organisms responds after the perturbation. The rate of response and the trajectory of how the distribution responds is a measure of resiliency and whether the assemblage shifts to a new baseline [48]. Here, we focus on three possible scenarios post-perturbation. In (*b*), the perturbation reduces the number of smaller individuals, as reflected in a reduction in the slope or exponent (*λ*). With time, species and individuals grow, reproduce, and interact with each other. As a result, the assemblage returns to a similar distribution, indicating that the size-dependent growth and mortality functions reach an approximate demographic steady state. However, in (*c*), the perturbation shifts the distribution, and over time, the assemblage does not return to the same baseline distribution but instead sees a shift in *m*_max_ and *λ*. In this scenario, this assemblage is less resilient than in scenario (*b*) as the resulting distribution shifts from the baseline. In (*d*), the perturbation mainly influences the largest individuals *m*_max_ compared to (*b*). Over time, the assemblage returns to the baseline as individuals grow in size.

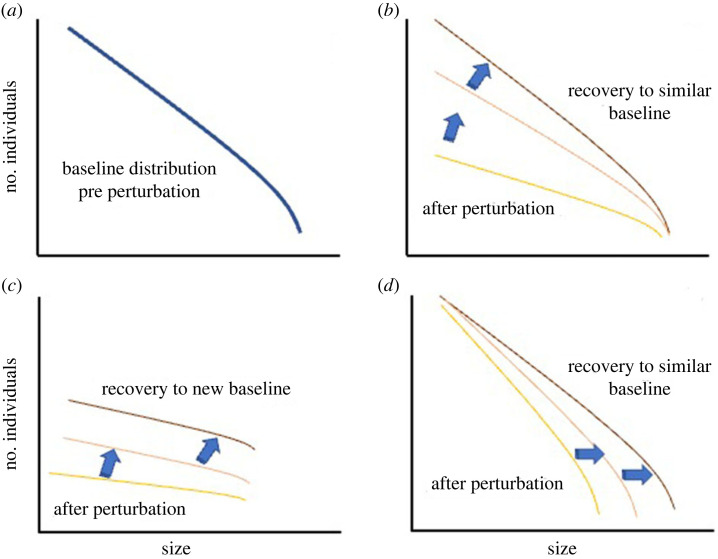

This equation indicates that if disturbance and external sources of mortality are low in populations, we expect to see a stable age or size structure that balances size-dependent growth and mortality [87,121,122]. The result is a characteristic scaling function—or the age or size distribution—called size spectra *f*(*m*) (boxes 1 and 2), where the ratio of mortality to growth across different sizes is bound by the −2 slope of the size distribution's logarithmic scale.

### (d) Disturbance, birth and death processes and resilience

Given our three above assumptions, resilience is not static but a dynamic process of disruption, adjustment, adaptation and eventual re-stabilization to a steady state where ‘birth and death’ rates equilibrate under resource (demographic) steady state. When a disturbance strikes, it disrupts the approximate symmetry of birth and death processes and disrupts energetic equivalence by freeing up resources. These shifts introduce asymmetry in birth and death rates into a transient (i.e. relatively short-term) phase, where dynamics depart from the asymptotic trajectories under a steady state [51,123]. The implication is that disturbance events shift the ‘birth and death’ processes and remove the assemblage from the resource steady-state until a new steady-state equilibrium emerges [48]. In the case of a disturbance (say a plant pathogen) that does not cause 100% mortality, we expect the new size structure to mirror a new demographic equilibrium because the generic symmetry principles of resource steady state and allometry symmetry will still hold, as presumably was true for the American chestnut [23]. The asymmetry caused by a rapidly shifting climate and/or disturbance may manifest as rapid species decline, differential mortality of larger individuals, loss of genetic diversity, or shifts in ecological roles, leading to a shorter-term period of instability and transition within the assemblage.

With a given disturbance or an uptick in environmental unpredictability, natural selection favours traits of individuals characterized by heightened resistance. The resilience of populations hinges on their inherent strategies and life histories. Notably, traits that mitigate short-term population declines or foster rapid post-disturbance are advantageous, surpassing traits optimized for sustained, long-term growth [98,124,125]. Resistance, in this context, refers to an individual's capacity to withstand disturbances without significant reductions in performance. Concurrently, an attribute like compensation, defined as the ability of a population to expand post-disturbance, becomes increasingly critical. Moreover, the duration of recovery, which is the time a population requires to revert to its equilibrium state after a disruption, assumes a pivotal role [70,126]. In essence, populations and species assemblages with traits that enhance resistance, promote rapid compensation, and minimize recovery time will probably exhibit heightened resilience in the face of environmental stochasticity.

Over time, selection pressures, ecological interactions and environmental conditions act upon the disturbed system, guiding it towards a new resource steady state and demographic equilibrium [1]. This adaptive phase is characterized by the emergence of new life-history strategies and the selection of traits better suited to the new conditions. As local processes take root, a new form of symmetry can emerge, reflecting the system's resilience. This return to a new symmetry may not be a return to the pre-disturbance state but a reorganization and realignment of birth and death processes, species traits and ecological roles under shared metabolic scaling rules and resource steady state that conform to the new environmental realities.

### (e) Empirical support

We point to several studies that broadly support the above predictions.

One way to investigate the properties of ecological interaction networks is to perturb a community by removing one or more species and monitoring the macroecological response as the network of species interactions is reorganized (box 1). A powerful example is an ecological experiment detailed by Brown [127]. Brown describes the work of PhD student Edward Boyer, from the University of Arizona [128], who studied the effects of the natural disappearance of a top predator from the northern Gulf of California (figure 3) on the community abundances of species. The large starfish, *Heliaster kubiniji,* was a major consumer of the sessile organisms (animals and algae) that compete for space on the rocky intertidal shore. We know from the classic ecological work of Paine that *Heliaster* is a ‘keystone’ species that regulated the abundance and utilization of space by the other species in this community [129,130]. This would predict that when *Heliaster* disappeared, the barnacles—the superior competitors—would increase and monopolize a larger share of space.

In 1978, *Heliaster* was decimated by the outbreak of a microbial pathogen and nearly went extinct throughout its geographical range in the northern Gulf. Boyer, who was already studying and surveying the organisms on rocky surfaces before the disappearance of *Heliaster*, continued his study to obtain a record of the changes caused by this natural disturbance. Figure 3 summarizes his findings. His work showed the division of resources and space among species on the rocky shore. These interactions result in an ‘emergent’ community distribution of abundance or dominance. Although this abundance or rank distribution was disrupted temporarily when *Heliaster* first disappeared, the distribution established rapidly even though *Heliaster* remained absent (figure 3). Notably, the relative dominance of particular species (indicated by their rank, not shown explicitly in figure 3) differed before and after the perturbation. These results are consistent with the prediction that the underlying birth and death processes were disrupted with the arrival of the novel pathogen. However, as time passed, the starfish remained absent, and the distribution of abundances among species returned to an approximate steady-state distribution (a return to a baseline macroecological pattern of abundance distribution), indicating a return to a symmetry of per capita species rates of birth and death.

Other studies that report supporting findings stem from the focus on species abundance distributions as a tool to assess impacts. In the 1970s, marine ecologists initiated inquiries into the potential of species abundance distributions (SADs) to serve as reliable indicators of environments disturbed by human activities, especially pollution [131–133]. Gray, in 1979 [134], investigated how various pollutants, including organic waste, oil and toxic industrial effluents, impacted and shifted the SAD and how removing these disturbances can result in the recovery of the original SAD. Several studies have shown how focusing on SADs is a useful tool to quantify the effects of disturbance more generally on communities [51,135,136]. In assessing the impact of disturbance and management interventions on biodiversity, the complete SAD offers a more comprehensive overview of ecological community attributes than basic diversity indices [136]. The SAD can serve as an effective early detection mechanism for disturbance effects on ecological communities and presents a user-friendly approach for evaluating the effectiveness of ecological management strategies [136].

**Figure 2.**
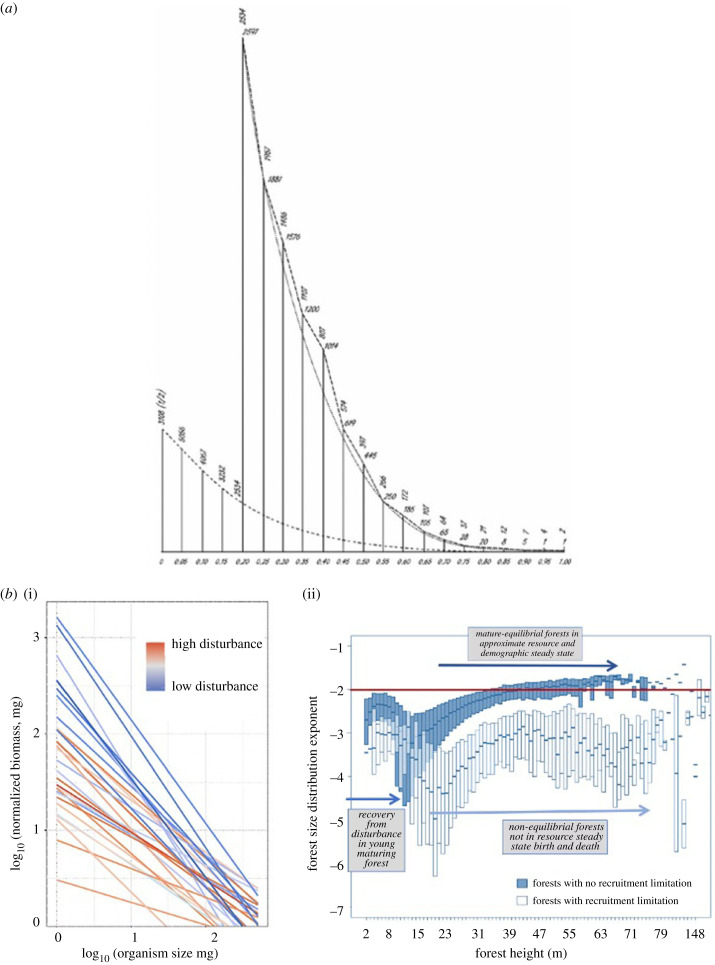
(*a*) From de Liocourt's 1898 paper showing the frequency distribution of tree sizes. The typical ‘J-curve’ indicates an abundance of small trees and very few large ones. Here, shows the mean number of trees in a healthy forest (solid line and horizontal numbers) and his ‘fitted distribution’ (dotted line and vertical numbers) of tree sizes in a disturbed forest. The *x*-axis legend is the diameter (cm), and the *y*-axis legend is the frequency. de Liocourt argued that the departure from the solid line could measure the impact of disturbance on forests. Thus, the impact of disturbance on forests could be measured by the deviation from the solid line to the dotted line. In this case, the disturbance decreased the abundance of the smallest trees. He hypothesized that as the forest recovered from disturbance, the forest would gradually return to the previous distribution (the solid line). As undisturbed forests across the globe tend to follow similar size distributions, more quantitative measures of the size distribution and their departures from the global mean and that predicted from steady state dynamics indicate a departure from steady state. (*b*) (i) Patterns in the size spectrum of 30 subtropical stream communities along a land-use gradient for aquatic macroinvertebrates along a broad land-use intensification gradient (from Atlantic forest to mechanized agriculture) in 30 Brazilian streams. The fitted power laws are plotted using each stream's intercept and slope values. The colour gradient is the degree of human impact from agriculture. The red colour indicates the highest level of impact from agriculture. Blue lines represent streams with the lowest human disturbance. Land-use intensification decreases ecosystem stability and enhances vulnerability to population extinctions by reducing the possible energetic pathways while improving efficiency between the remaining food-web linkages. Theory predicts that for size spectra in undisturbed trophic networks the exponent should begin to approximate −1 as is observed here. Data and figure redrawn from Collyer *et al*. [137]. (ii) Forest tree size distribution recovering from disturbance from approximately 100 000 forests plots in North America spanning disturbance gradients. The plot shows scaling exponents in plots with (blue boxes) and without apparent recruitment limitation (clear boxes), shown as a function of forest height (a proxy for time since disturbance). The red line is the theory prediction from metabolic scaling theory (MST) of the size distribution exponent under resource and demographic steady state where births and deaths are equal. By contrast, forests with a dearth of small stems owing to external disturbances (fire, climate extremes, excessive size-dependent herbivory etc.) exhibit recruitment limitations and resource steady state that is not reached. These non-equilibrial forests are characterized by a different allometric scaling of birth and death rates. Therefore, the idealized conditions required for MST will not hold and the observed scaling relationships will differ owing to the differing birth and death rates from those rates under resource steady state. Data and figure redrawn from Duncanson *et al*. [16].

Several studies also report results consistent with the use of size distributions to assess the impact of disturbance. Recent work by Collyer *et al*. [137] (figure 2*b*) assessed the relationship between macroinvertebrate size structure along a human land-use gradient (a surrogate for increasing perturbation and disturbance) in subtropical Brazilian streams. Consistent with expectations in box 2, they found that the size distribution is characterized by an approximate inverse power law with many small individuals and few large (figure 2*b*). The scaling of size spectra shifted with disturbance from land-use intensification. Streams in increasingly more disturbed agricultural watersheds had more shallower slopes and lower intercepts when compared to undisturbed streams. Further, increasing land-use intensification decreased the size range of organisms. Disturbance decreases ecosystem stability and enhances vulnerability to population extinctions by reducing the possible energetic pathways while improving efficiency between the remaining food-web linkages.

**Figure 3.**
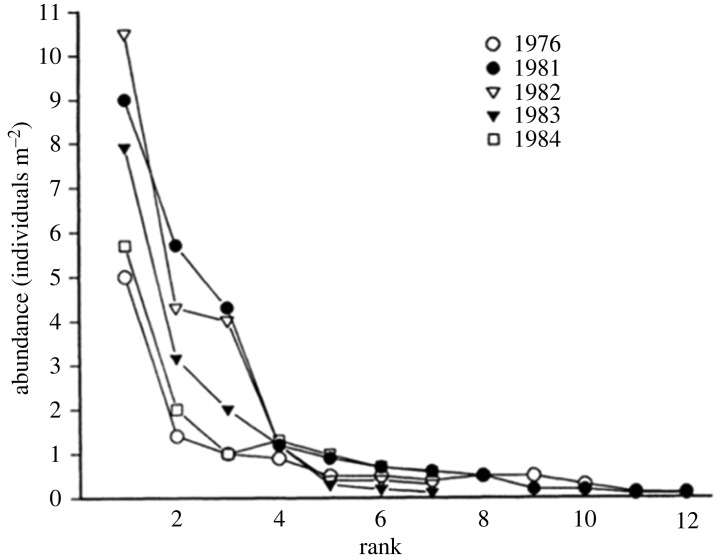
The resilience of an intertidal community following the perturbation of a novel pathogen as viewed through the dynamics of species abundance distribution within a community. This ‘dominance-diversity’ relationship shows the ranked relative abundances of species on the shore in the intertidal of the northern Gulf of California and the near extinction of the top predator, the starfish *Heliaster kubiniii*, in 1978. Compare the dominance-diversity relationship pre-pathogen in 1976 to post-local extinction of the top predator in 1981 and the ‘recovery’ of the community up to 1984. Note that immediately after the disappearance of the starfish, the community became dominated by a few species, as predicted by Robert Paine's keystone predator hypothesis. However, as time passed, the starfish predator remained absent. In this community, a diverse carnivore guild with a high degree of dietary overlap with *Heliaster* appears to have compensated for the absence of *Heliaster* predation in controlling prey densities. The distribution of abundances among species returned to the original values indicating a return to a baseline macroecological pattern of abundance distribution even though the species composition had changed. Graph redrawn from Boyer [128].

Second, Enquist & Niklas [76] and Duncanson *et al*. [16] analysed size distributions across diverse forest communities. They found that although size distribution exponents varied [76], they clustered around the steady state prediction of –2, in general agreement with the EER prediction (see figure 2*b*; figure 3). Further, variation in the exponent was biogeographically structured, with more disturbed forests showing more deviations from the steady state −2 exponent.

Third, similar support comes from Kerkhoff & Enquist's study of an old-growth *Pinus ponderosa* forest in northern Arizona, documented since 1920 [138], which revealed changes in forest size structure owing to human interventions [48]. Historically shaped by frequent disturbance low-intensity fires that promote an open mosaic of mature trees, the forest's dynamics shifted when the twentieth-century brought suppression of grazing and fires. This led to sporadic *P. ponderosa* recruitment and a shift towards catastrophic crown fire regimes [139]. After fire suppression was stopped, the forest's size structure steepened, deviating from earlier patterns where a bimodal distribution suggested fire-driven recruitment limitations. Specifically, pre-suppression records in 1920 showed a notable gap in the 20- to 40 cm size range, probably tied to the ground fire return interval. Post-suppression, the gap closed, indicating a more continuous size distribution and marking a significant change in the forest's structural dynamics. This shift from scaling and the observed structural gap in 1920 highlights a regime shift from ground fire as a natural structuring process influencing recruitment and forest architecture.

Lastly, we point to several studies from botanists and forest ecologists that have turned to de Liocourt's law for a helpful definition of a ‘healthy’ sustainable forest ecosystem [61,140]. Precise measurement of the effects of emerging pathogens is critical for crafting effective management strategies [140]. In the context of novel fungal forest pathogens, tree mortality is the primary metric for assessing loss [7]. However, since tree mortality is a natural aspect of forest dynamics, establishing a reference point to measure the additional mortality attributed to new pathogens is a significant hurdle.

In the context of forest and ecosystem ‘health’, introducing a new pathogen can lead to rapid changes in the host population. These changes become apparent when the pathogen elevates mortality rates or disrupts birth and death rates, deviating from the established norms observed in the pre-pathogen forest conditions. To assess a pathogen's impact, researchers can establish a baseline mortality rate using a de Liocourt curve generated from comprehensive tree inventory data. Changes in the host population would occur if the new pathogen caused higher mortality or changes in birth or death rates beyond those found under pre-pathogen conditions in the forest. Foresters can obtain baseline mortality from a de Liocourt curve calculated from tree inventories carried out over large areas to quantify the impact of a pathogen [61]. In essence, variations in size distributions offer a robust and comprehensive metric for correlating pathogen effects with fundamental biological processes like death, growth and birth—principles that are universally applicable to all living organisms.

Any level of biological organization discussed in this chapter can be understood as a network of energy, matter and information transfer among entities whose structural and dynamic properties may provide baselines to assess system resilience. Abundant empìrical and theoretical evidence has shown that the scaling of the number of components, their connectance, interaction strengths and other topological characteristics (degree distribution, modularity and nestedness), as well as patterns in species trait (i.e. body size), affect network stability and resilience (e.g. [141–145]) and that several of these characteristics have been present from deep-time (Cambrian) interacting networks [146]. Taken altogether, these results suggest that the space of possible network organizations and reorganizations under disturbances is restricted to a relatively small subset of configurations that confer some notion of stability and resilience, and, ultimately, persistence. The most basic network model, where the probability of interaction *C* between *S* species and the strengths of interaction are assigned at random [147,148], shows that large networks of interacting species will be stable only if

3.10 $$\frac{1}{\sqrt{CS}}>\alpha .$$

From this simple beginning, it has been shown that non-randomness in network topology and interaction strengths affect persistence and allow for the resilience of large networks [142,144]. As noticed by Busiello *et al*. [149], if networks are sparse such that *C* proportional *S*^−1^, then their stability will not be affected by their size as predicted by May [148] but instead will show resilience, reorganizing and exploring alternative stable states (also known as dynamic robustness). Interestingly, empirical evidence shows that biological networks are indeed sparse [149]. Scaling network properties may provide an important foundation for building a predictive ecological resilience theory.

Finally, it is important to mention that several other scaling relationships in complex ecological systems could be important to assess persistence and resilience (see reviews in [71,150,151]). Notable among them is the scaling of biodiversity fluctuations and spatial patterns. Examples of the scaling of fluctuations (typically in migration or speciation and extinction, [152]) are the characterization of biodiversity collapses in introduced island biotas [153] in the context of the self-organized criticality theory [154] and the fossil record [155]. Scaling patterns in the spatial attributes of landscapes (connectivity and patch size distributions) are related to shifts between alternative states and can be used as early warning signals of systems undergoing ecological transitions (e.g. [156,157]) and quantify deviations from an undisturbed state. Kéfi *et al*. [158] have shown that the patch size distribution of unperturbed Mediterranean ecosystems follows a power-law distribution. Still, as grazing pressure increases, the distribution deviates from a power-law, which indicates a transition to a desertic state. Similar results are associated with the spatial pattern of habitat in relation to the overall connectivity of a landscape, showing the existence of a percolation threshold associated with a marked transition in connectivity that might affect species persistence [156,159,160] and the spatial phase transition between forest and savannahs, which Abades *et al*. [161] hypothesize results from a percolation threshold in grass cover linked to the emergence of a spatially connected or spanning cluster of grass that facilitates fire spread.

## 4. Discussion

### (a) de Liocourt's law of resilience reflects compensatory biological dynamics

How does the biotic world respond to climatic and biotic change? In the face of environmental change, biology responds—organisms often compensate, acclimate, adapt, and change the nature of their ecologies [162,163]. The invasion of a pathogen, the loss of a predator and the arrival of a new competitor or mutualist change the calculus of interactions and the impacts on fitness within and across taxa.

Recent studies indicate that compensatory dynamics can enhance ecological resilience in the face of perturbations, provided a ‘reservoir’ of rare phenotypes and potential ecological interactions is available [164–166]. Compensatory dynamics involve changes in one species' population being offset by changes in others. This balance can arise from colonization-extinction dynamics or adjustments in resident species' populations owing to changing resources [98]. If local colonization offsets local extinctions, then relatively high-magnitude changes in species richness and the rank-abundance distribution may be rare (e.g. [51,167,168]). The ability of ecosystems to exhibit compensatory biodiversity responses is likely to depend on the overall number and magnitude of perturbations and the environmental setting relative to the traits and life-history strategies present. Environmental perturbations can be either buffered or amplified by biodiversity depending on characteristics of local ecosystems, such as the magnitude of the changes and their effects on dominant species. Perturbations are likely to have greater impacts when the dominant species are near the limits of their distributions and already under stress.

Similarly, diverse species traits (increase in trait variance) within a community may allow population-level compensatory dynamics to maintain similar ‘birth and death rates' and, hence, similar abundance-related community-level properties under changing environmental conditions [116,167,169]. Here, reorganization at the level of individual species' populations did not result in equivalently large-magnitude changes in community-level properties. Several additional studies have documented compensatory dynamics in community-level properties and have indicated that compensatory dynamics may be an essential mechanism for mitigating the effects of disturbances [168,170,171], but see Elmendorf & Harrison [172].

### (b) Developing a general ecological or system resilience theory based on scaling is critical

One challenge in assessing and expanding our arguments is understanding how spatial and temporal scales influence the consistency of ecological relationships. This complexity is evident in historical examples where such relationships have become uncoupled following major extinction events. For instance, the connection between species diversity and ecological function was disrupted over extended time frames after the end-Cretaceous mass extinction [173]. This event also led to a disconnection between body size and brain size in Cenozoic mammals [174]. Although these instances of decoupling present challenges—it is noteworthy that, despite extensive research on species abundance distributions, body size distributions and other key macroecological patterns—few ecologists have used these robust patterns as quantitative benchmarks. These benchmarks could be instrumental in measuring the impacts of environmental disturbances or in assessing ecosystem resilience.

Another challenge is that in the Anthropocene, ecological systems confront directional climate changes, escalating variability, and the emergence of novel climates [32,175] and ecological interactions, which in turn affect birth–death dynamics. The nature of rapid changes in the Anthropocene challenges the applicability of the proposed approach to increasingly disturbed systems (see [176,177]). A challenge lies in distinguishing between trends stemming from sustained directional changes and escalating disturbances from climate and land use change and those arising from temporary fluctuations. Nonetheless, we believe the arguments advanced here provide the basis for the next steps, including clarifying how macroecological expectations, including those related to community size distributions (size spectra) and community rank-order structure, can be analysed to effectively discern and integrate the diverse types and timescales of these ecological forcings.

The size and species abundance distribution measurements are often straightforward and hold immense promise as diagnostic tools. They could significantly enhance the quantitative and predictive aspects of adaptive management in ecology. Their underuse represents a missed opportunity for employing established ecological patterns in practical, management-oriented applications [48,136]. Size distributions, scaling relationships and species abundance distributions all have enormous potential to serve as environmental indicators indicative of the state (health) of an ecosystem [112,178]. Deviations from scaling relationships and other macroecological patterns can be used as a signature of specific underlying structuring processes—such as the impact of a pathogen—or may indicate the transient reorganization of the system.

## 5. Conclusion

The parable of the North American chestnut underscores the challenges of predicting ecosystem responses to major disturbances. We have focused on the increasing impact of human-induced pandemics in the plant world and their cascading ecological impacts. Building on previous work on resilience and scaling, we have argued that ecologists need to move beyond the ‘ball and cup’ analogy typical of our field and move towards more formal quantification of the cascading impacts of disturbances and perturbations via measures of resilience that are framed at a more systemic level (such as scaling or symmetric distributions) that are still quantitative. Here, we have argued that more systemic, quantitative measures naturally emerge if we embrace a more macroecological and dynamic scaling view of resilience. To cite Falk *et al*., ‘resilient responses are emergent properties resulting from component processes of persistence, recovery and reorganization, with different mechanisms at work in each mode’ [1, p. 1]. This paper advocates for a diffusion and scaling approach to resilience, contrasting it with the dynamical systems (ball and cup) approach. The former is easier to measure and rests on a well-developed theory grounded in fundamental principles. By contrast, the later approach poses challenges in measurement and detection, often requiring a posterior analysis.

A theoretical foundation for resilience is anchored in three insights: (i) the processes of birth, death, and demographic, resource, or energetic symmetries within ecological systems form a foundation for characterizing ecological dynamics; (ii) ‘birth and death processes’ themselves often encapsulate and reflect these underlying biological symmetries, via a balance in the rates and patterns of species turnover, proliferation and demise; and (iii) biological scaling laws profoundly influence how these processes scale with a key trait, organismal body size, and ramify across different levels of ecological organization.

A growing body of literature demonstrates that a general theory—based on a diffusion approximation and metabolic scaling that centres around macroecological patterns such as abundance distributions and size spectra in ecological assemblages—can establish essential baselines for resilience assessment. Specifically, de Liocourt's law for forest size distributions provides an example of a dynamical emergent baseline originating from ecological processes of allometric growth and steady-state birth and death rates. Nonetheless, other macroecological patterns could probably be used as baselines (abundance distributions, species-area curves, ecological interaction networks etc.). Building on de Liocourt's law, we outlined several steps to develop a general ecological or system resilience theory, and we show how several recent studies have applied parts of this approach to quantify the impact of pathogens and other perturbations on ecological systems.

We conclude with the following points.

(i) Understanding responses to perturbation: we focus on how ecological systems respond to perturbations, analysing changes in ecological interactions, energy flows and material exchanges to determine if the system returns to a similar ecological structure.
(ii) Scaling and macroecological approach: we advocate for a scaling and macroecological approach to establish quantitative baselines for assessing ecological resilience. The rise of disturbances in the Anthropocene challenges conventional notions of disturbance and resilience in ecological science.
(iii) Compensatory dynamics: ecological systems exhibit multiple routes of compensation in response to environmental changes. Compensation is reflected by shifts in ‘birth and death’ processes. Evaluating emergent macroecological patterns and scaling properties during perturbations provides a powerful, underused approach. These examples highlight simple underlying processes influencing complex organizations.
(iv) Foundations for a general theory: the foundations for a comprehensive ecological resilience theory already exist. Insights from François de Liocourt's ‘inverse J-shaped’ frequency distribution of organism sizes highlight the presence of birth–death equilibria, forming a baseline for measuring disturbances in ecological systems. The Chapman–Kolmogorov equation and metabolic scaling theory provide a theoretical foundation for resilience studies. It allows us to formulate a theory to predict future system states based on current conditions and transition probabilities by focusing on birth and death processes. By integrating biological scaling and resource and demographic steady state, we gain deeper insights into population dynamics and trajectories of change. A holistic understanding of ecosystem resilience emerges through the fusion of macroecology, mathematical models and population biology, guided by these historical theories. These insights equip us to predict and manage future ecological challenges.

A signature of the Anthropocene is one of increasing perturbations [179]. There are several promising approaches that ecologists can use to understand better and predict how ecological systems respond to increasing disturbance [38,127,134]. Analysing body size distributions and abundance across space and time offers key baselines to quantify perturbations and measure resilience. The examples discussed in this paper indicate that patterns repeatedly emerge in different kinds of organisms from different environments. The focus of macroecology on recurring patterns across various organisms and environments reveals straightforward processes that often shape the seemingly complex organization of ecosystems. Such processes have far-reaching effects on emergent dynamics, establishing a solid foundation for evaluating ecological resilience in our deeply interconnected biosphere.

## Data Availability

This article has no additional data.
